# Internal structure and reliability of the *Attachment Insecurity Screening Inventory* (AISI) for children age 6 to 12

**DOI:** 10.1186/s12888-018-1608-z

**Published:** 2018-02-05

**Authors:** Anouk Spruit, Inge Wissink, Marc J. Noom, Cristina Colonnesi, Nelleke Polderman, Lucia Willems, Charlotte Barning, Geert Jan J. M. Stams

**Affiliations:** 1 Research Institute of Child Development and Education, University of Amsterdam, Nieuwe Achtergracht 127, 1018 WS Amsterdam, The Netherlands; 2 William Schrikker Group, Bijlmerdreef 101, 1102 Amsterdam-Zuidoost, The Netherlands; 3 Basic Trust, Postbus 43, 4300 AA Zierikzee, The Netherlands; 4 Zaanstad Medical Center, Department of Psychiatry, Kon. Julianaplein 58, 1502 DV Zaanstad, The Netherlands

**Keywords:** Attachment, Assessment, Middle childhood, Parent report, AISI

## Abstract

**Background:**

The aim of the present study was to examine the internal structure and reliability of the Attachment Insecurity Screening Inventory (AISI) 6–12. The AISI 6–12 years is a parent-report questionnaire for assessing the parents’ perspective on the quality of the attachment relationship with their child aged between 6 and 12 years.

**Methods:**

The sample consisted of 681 mothers and fathers reporting on 372 children (72.3% adoption parents, 14.9% non-biological primary care takers including foster parents, and 12.8% biological parents). The internal structure was assessed with multilevel confirmatory factor analyses (CFA) and the reliability of the scores with Cronbach’s and ordinal alphas.

**Results:**

Multilevel CFA confirmed a three-factor model of avoidant, ambivalent/resistant and disorganized attachment. Multi-group CFA indicated full configural and metric measurement invariance, and partial scalar and strict measurement invariance across mothers and fathers. Reliability coefficients were found to be sufficient.

**Conclusions:**

This study showed the potential of using parental reports in the initial screening of attachment related problems, especially considering the practical approach of parental reports. However, further development of the AISI 6–12 years seems important to increase the validity of the AISI 6–12 years. In addition, future studies are necessary to replicate the current findings, and to strengthen the evidence that the AISI 6–12 years is appropriate for the use in middle childhood and validly assesses the parents’ perspective on attachment insecurities in their child.

**Electronic supplementary material:**

The online version of this article (10.1186/s12888-018-1608-z) contains supplementary material, which is available to authorized users.

## Background

How we attach to significant others is a life-course developmental phenomenon of stability and change, which not only shapes who we are in our relationships with others, in particular those with whom we have an enduring emotional bond, but also shapes social development [1, 2]. In general population samples, approximately two-thirds of young children derive major support from relationships with their parents who are perceived as both a secure haven and secure base, and are therefore designated as securely attached children [2, 3]. Children with insecure attachments are children who do not perceive their caregiver(s) as a secure haven and secure base and cannot find the balance between proximity and distance to an attachment figure, or even have no strategy at all to keep such a balance (i.e., disorganized attachment) because the attachment figure is a source of fear and discomfort that should be controlled. These insecurely attached children have a greater risk for psychopathology, such as internalizing problems (anxiety, depression) or externalizing problems (aggression, delinquency) [4–6]. Therefore, it is of great clinical and scientific importance that insecure child-parent attachment relationships be validly and reliably measured.

Even though attachment is considered to be a life-course phenomenon, most researchers on attachment have focused their attention on early childhood, adolescence or (young) adulthood, but less on middle childhood [7]. Several valid and reliable instruments, of which some are even considered to be golden standards, have been developed to assess attachment in the first years of life and beyond the age of 12, such as the Strange Situation procedure in infancy [8], the Attachment Q-Sort in toddlerhood [9], and the Adult Attachment Interview in adolescence and (young) adulthood [10]. Scholars have developed instruments to assess attachment in middle childhood (i.e., children from age 6 to 12), such as the self-report People in My Life-scale [11, 12], narrative storytelling assessments, and observational instruments [13]. While it is often assumed that attachment is best assessed by means of behavioral observation in early childhood, or alternatively projective measures in toddlerhood (e.g., doll play), and in-depth interviewing during adolescence and (young) adulthood by means of representational measures, it is not yet clear how attachment in middle childhood should be assessed [7].

Recently, it has been argued that there should not be a dominant measurement approach, and the question should not be “what is the golden standard”, but “which component or aspect of the attachment construct is measured” ([7], p. 9). We would like to add ‘for which purpose’, for instance, scientific research on attachment-related developmental processes, clinical practice to guide attachment-based intervention targeting insecurely attached children and their parents, or both. Attachment may not only be assessed in terms of secure or insecure attachment behaviors or representations, focusing on the internal working model of attachment [13], but also from the perspective of the child or parent. It is important that instruments are specific about the attachment components they measure, and about the purposes for use.

Additionally, we feel the need for straightforward, practical, and economical instruments to assess the quality of attachment relationships between parents and their child. Currently available instruments, such as observational instruments, projective doll play interviews, and self-report interviews are rather time consuming, require extensive training, or are less suitable for children who have limited self-refection abilities [14]. In clinical and scientific practice, where money and time are important factors, the lack of straightforward instruments could lead to the decision to not assess the attachment relationships of children. It is interesting to test whether parental reports on attachment relationships could fill this gap.

Polderman and Kellaert-Knol [15] developed the Attachment Insecurity Screening Inventory (AISI) 2–5 years as a clinical tool to be used in attachment-based intervention, and it has also been used for scientific purposes [16]. The AISI 2–5 years is a brief 20-item parent self-report measure in Dutch, assessing the parents’ perception of the quality of the attachment relationship with their child, in particular focusing on insecurity, which was recently validated [17]. The AISI 2–5 years has not been validated in other countries. The AISI 2–5 years [15] showed a sufficient 3-factor model fit, measurement invariance across mothers and fathers and across the general and clinical population, good internal consistency, and indicators of concurrent and convergent validity [17]. This instrument can be used for the initial screening of attachment related developmental problems in children. The current study examined the internal structure and reliability of the middle childhood version of the AISI [18] in a clinical sample of children aged 6 to 12, primarily from adoptive or foster care families, as these children are at risk of experiencing attachment-related problems [19–21].

The AISI 2–5 years [15] assesses parents’ perception of Insecure-Avoidant (Type A), Insecure-Ambivalent or resistant (Type C), and Insecure-Disorganized (Type D) attachment relationships with their child. Insecure-Avoidant children (Type A) minimize their attachment behaviors, which is an insecure but still organized strategy to keep proximity to a consistently insensitive and possibly rejecting parent [8]. Insecure-Ambivalent children, also designated as Resistant or Preoccupied children (Type C), maximize their attachment behaviors, which is an insecure-organized strategy to keep proximity to a parent who is inconsistently sensitive [8]. Insecure-Disorganized children (Type D) do not have an organized strategy to keep proximity to their caregiver, and they may use controlling strategies, such as disorientation, withdrawal, and high intrusiveness, in response to harsh parenting or disrupted parental behaviors [22]. These controlling behaviors may be punitive and aggressive or care giving (being overly solicitous and nurturing with the parent) in order to guide the parent’s behavior [23, 24].

The AISI 6–12 years [18] assesses the same insecure attachment dimensions as its precursor, the AISI 2–5 years, with the same set of 20 items, although some items have been slightly changed for reasons of age-appropriateness [18]. Although social networks, involving peers and teachers, become significantly larger for children during middle childhood compared to early childhood, parents remain the primary attachment figures [25, 26], which legitimates the idea that (the measurement of) attachment to parents is still an important issue at this age. However, the literature also points at qualitative differences in attachment of children in the age of 6 to 12, compared to 2 to 5 year olds. Bosmans and Kerns [7], for instance, note that short separations from parents become less stressful and problematic in middle childhood, and no longer elicit the immediate need for proximity. They emphasize that the goal of the attachment system gradually changes from proximity to the attachment figure to availability of the attachment figure in the transition from early to middle childhood [7]. In addition, new situations that may elicit support-seeking behavior emerge in late middle childhood, such as academic failure and social conflict [7, 27]. Moreover, the attachment behavioral system becomes more sophisticated, as children are becoming increasingly able to regulate emotions and communicate about emotions, plan and organize behavior, and understand the difference between their own perspective and that of others [28]. Therefore, it is important to examine whether the AISI validly and reliably assesses the parent’s perception of the quality of attachment relationships for 6 to 12 year olds.

The aim of the present study is to examine the internal structure and reliability of the AISI 6–12 years in a clinical sample of adoptive, foster care, and biological families with children aged 6 to 12, by means of a (multilevel) Confirmatory Factor Analysis (CFA) and the computation of ordinal and Cronbach’s alpha coefficients as measures of internal consistency reliability. A three-factor structure consisting of avoidant, ambivalent and disorganized attachment was expected. Psychometric characteristics of the AISI 6–12 years have not been evaluated before. In addition, we aim to test the measurement invariance (i.e., whether the perception of the attachment relationship is measured the same for parents of different sexes) of the AISI 6–12 years for mothers and fathers raising the same child.

## Method

### Participants

Participants were 681 Dutch parents (72.3% adoption parents, 14.9% non-biological primary care takers including foster parents, and 12.8% biological parents) of 372 children (57.4% boys and 42.6% girls) aged 6 to 12 (*M* = 9.04; *SD* = 1.89). The sample consisted of 309 mother-father dyads in which both parents reported on their child, and 51 mothers and 12 fathers who solely reported on their child. The mean number of children per family was 2.35 (*SD* = 1.25, range 1 to 8). The children in the study were first children in 50.3% of the cases, and 30.7% second children, 8.5% third children, and the remaining 10.5% the fourth, fifth, sixth, seventh or eighth child. Mean age of the mothers was 43.43 years (*SD* = 5.88, range 23 to 62) and fathers 45.31 years (*SD* = 5.08, range 22 to 66). A total of 5.8% of the mothers had lower levels of educational attainment (i.e., low vocational training), 34.9% had middle levels of educational attainment (i.e., middle vocational training), and 59.3% had higher levels of educational attainment (i.e., higher vocational training or university). For fathers, this was 9.0, 34.1, and 56.9% respectively.

### Procedure

The families were referred to the attachment-based Basic Trust intervention (see [16]), because of children’s emotional and conduct problems, and the suspicion of underlying attachment problems. Basic Trust is a group practice with therapists providing attachment-based intervention by means of videofeedback and parenting advice, designated as the Basic Trust Method [16]. Attachment Insecurity Screening Inventory 6–12 years [18] was filled in by both parents as part of the intake procedure (before the start of the intervention), and provided informed consent for the present study. The parents did not receive a reward for their participation in the study.

### Measures

#### Attachment insecurity screening inventory 6–12 years

The AISI 6–12 years [18] contains 20 items in Dutch, asking parents how they perceive the attachment behaviors of their child, with a 6-point Likert-type response format: never, sometimes, regularly, often, very often, and always (with scores ranging between 1 and 6). The items belong to three subscales assessing: avoidant (range 8–48), ambivalent (i.e., resistant, dependent; range 7–42) and disorganized attachment (range 5–30) child-parent attachment relationships (see the Additional file 1: Supplementary file AISI 6–12). Subscale scores represent the sum scores of all items of the scale. The AISI 6–12 years is an adaptation of the AISI 2–5 years [15, 17]. Four items have been rephrased to better represent insecure child-parent relationships in middle childhood. Item 6 ‘*Does your child cling to you*?’ was changed into *‘Does your child always stay close to you?’*. Item 11 *‘Is it easy for your child to resume contact with you after you have been separated?’* was changed into *‘Does your child make good contact with you after you have been away for a short period of time?’.* Item 13 *‘Is your child excessively emotional when you leave him/her for a short period of time?’* was changed into *‘Does separation from you cause overly strong emotional reactions in your child?’.* Finally, item 15 ‘*Does your child want to be put down and then immediately picked up again?’* was changed into *‘Does your child want to be left alone and simultaneously seeks contact with you?’* [18]*.*

### Analyses

We first tested the internal structure of the AISI 6–12 years by means of a multilevel Confirmatory Factor Analysis (CFA) in Mplus [29] and R [30], as children from the same family (level 1) were nested in father-mother dyads reporting on the same child (level 2), implying a multilevel structure of the data. In addition, multilevel models have the advantage of using all the available data (including those from participants with missing data, in this study, the families with single parents) [31]. To examine the factor structure of the AISI 6–12 years, we followed the stepwise procedure of Muthén [32]. In the first step (model 1a and 1b), we provided initial information on the factor structure of the AISI, without taking the multilevel structure into account: model 1a tested the baseline model, while model 1b tested whether the model can be improved by removing problematic items (e.g., not allowing correlated measurement errors). In this adjusted model, items were removed when the factor loadings were below .30, if they did not significantly contribute to the factor solution (i.e., low R^2^ and/or high error variances), and if they showed many significant correlated errors with other items that could not be explained by (for instance) specific item content or similar wording [33, 34]. In the second step (model 2a and 2b), we examined the factor structure of the AISI by means of a multilevel CFA: model 2a was the (best fitting) model after having applied criteria for improvement of model fit based on modification indices (see below); model 2b examined whether further improvement of model fit could be obtained by allowing within factor measurement error correlations of items with comparable item content [33, 34].

Then, we tested the measurement invariance of the best fitting AISI 6–12 years model for mothers and fathers raising the same child in Mplus [29]. It was not possible to apply a multilevel model in the multiple group CFA to test measurement invariance across fathers and mothers, because the second level (father-mother dyad) identification number is not allowed to appear in both sex-groups in multilevel analysis. Therefore, when testing the measurement invariance across fathers and mothers, we proceeded with step 3 to 6, without taking into account the nested structure of the data. In step 3 to 6, we tested for configural (model 3), metric (model 4), scalar (model 5a), and strict (model 6a) measurement invariance of the best fitting factor model in a standard multi group CFA. If necessary, additional models could be tested to test partial measurement invariance, accounting for the possibility that only one or two (but not all) factors prove to be fully invariant [32, 35, 36]. The descriptives of the AISI 6–12 and the paired t-tests were calculated in SPSS [37].

Fit indices were used to test model fit in all models. The following cut-off values are indicative of good model fit: CFI > .95, TLI > .95, SRMR < .08, and RMSEA < .07 [38, 39]. A non-significant Chi-Square indicates exact model fit, and a ratio between the *X*^*2*^ statistic and the degrees of freedom (*df*) that is lower than 3:1 indicates a close fit to the data [38, 39]. Any significant decrease of model fit (based on a *Χ*^*2*^ difference test or a drop in CFI greater than .005) indicates that the more stringent condition of measurement invariance for that model has not been met [40, 41]. Reliability of the scores was tested by computing ordinal and Cronbach’s alphas for each subscale of the AISI 6–12 years in R [30], because of the ordinal 6-point response format [42].

## Results

### Internal structure

**Fig. 1:**
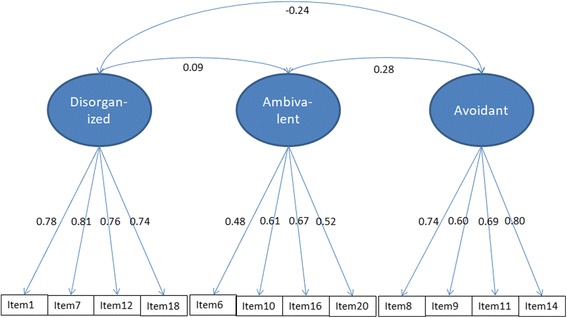
Model 1b: Correlations and Factor Structure

Table 1 presents the fit statistics of the different models that were examined. In the first step, we tested the baseline model (model 1a) including all 20 items of the AISI, which did not yield a satisfactory factor solution. However, model 1a could be significantly improved (*X*^2^(111) = 658.284, *p* < .001, Δ CFI = .087) by removing eight items reflecting ambivalent attachment (item 2, 13, and 15), disorganized attachment (item 4), and avoidant attachment (item 3, 5, 17 and 19). This resulted in model 1b, which yielded a good fit (see Fig. 1). The analyses were conducted on the entire sample (*N* = 681).

**Table 1:** Fit Statistics CFA Models (*N* = 681)

	*X* ^*2*^	*df*	*p*	*X*2*/df*	*RMSEA (90%CI)*	*TLI*	*CFI*	*SRMR (Within/Between)*
Model 1a	792.060	167	< .001	.743	.074 (.069;.079)	.864	.880	.076
Model 1b	133.776	51	< .001	.632	.049 (.039;.059)	.957	.967	.039
Model 2a	209.336	102	< .001	.052	.039 (N/A)	.936	.951	.056 / .084
Model 2b	165.701	97	< .001	.708	.032 (N/A)	.957	.968	.055 / .074
Model 3	174.059	102	< .001	.706	.046 (.034;.057)	.962	.971	.046
Model 4	181.464	111	< .001	.635	.043 (.031;.054)	.966	.971	.049
Model 5a	213.692	120	< .001	.781	.048 (.037;.058)	.958	.962	.051
Model 5b	184.181	115	< .001	.602	.042 (.030;.053)	.968	.972	.049
Model 6a	239.002	132	< .001	.811	.049 (.039;.059)	.957	.957	.055
Model 6b	198.931	126	< .001	.579	.041 (.030;.052)	.969	.970	.054

*Note.* Model 1a = the baseline model; Model 1b = model with problematic items removed; Model 2a = multilevel CFA; Model 2b = Model 2a when allowing within factor measurement error correlations of items with comparable item content; Model 3 = configural measurement invariance; Model 4 = metric measurement invariance; Model 5a = scalar measurement invariance; Model 6a = strict measurement invariance. Model 5b and 6b tested partial measurement invariance

In the second step, we tested whether the factor structure of model 1b would hold when taking the multilevel structure of the data into account. We examined all 20 AISI items by means of a multi-level CFA, which after model improvement resulted in the same set of 12 items as in model 1b, with substantial individual ICC ranging between .266 (item 16) and .562 (item 12), which supports the CFA multi-level approach. This more stringent test of the model yielded a good model fit on all the fit indices, except for the TLI and the between dyad level SRMR. Model 2b did significantly improve model fit (*X*2(5) = 43.635, *p* < .001, Δ CFI = .017) by allowing correlated measurement errors between the following pairs of items in the within dyad model (7 and 12, 8 and 9) and between dyad model (6 and 20, 7 and 18, 10 and 16). The results of the second step indicate that the factor structure presented in Fig. 1 holded in the multilevel analyses.

The subscale scores of the 12-item AISI 6–12 years correlated highly and significantly with the subscale scores of the 20-item AISI 6–12 years. For the Ambivalent scale, this was .924, for the Avoidant scale .935, and for the Disorganized scale .973. Table 2 presents the descriptives of the scales of the 12-item AISI 6–12 years.

**Table 2:** Descriptives of subscales of 12-item AISI 6–12 years

	Total sample (*N* = 681)		
	*M (SD)*		
Avoidant	16.966 (4.21)		
Ambivalent	11.167 (3.60)		
Desorganised	12.013 (4.40)		
Total insecurity	40.147 (7.18)		
	Mothers (*n* = 361)	Fathers (*n* = 320)	
	*M (SD)*	*M (SD)*	*t* ^*a*^
Avoidant	17.031 (4.42)	16.894 (3.98)	− 0.425
Ambivalent	11.609 (3.77)	10.669 (3.34)	−3.457**
Desorganised	12.501 (4.62)	11.463 (4.08)	−3.115**
Total insecurity	41.141 (7.22)	39.025 (6.97)	−3.880***
	Non-biological parents (*n* = 594)	Biological parents (*n* = 87)	
	*M (SD)*	*M (SD)*	*t* ^*b*^
Avoidant	16.931 (4.23)	17.207 (4.14)	− 0.729
Ambivalent	10.958 (3.44)	12.598 (4.27)	−3.843***
Desorganised	11.874 (4.28)	12.966 (5.07)	−1.875
Total insecurity	39.763 (6.95)	42.770 (8.12)	−3.559***

*Note.* ^a^Independent *t*-test. ^b^*t*-test of multilevel model. *p < .05; **p < .01; ***p < .001

Mothers reported significantly more attachment difficulties on the Ambivalent, Disorganized, and Total Insecurity scales than fathers. Biological parents reported significantly more attachment difficulties on the Ambivalent and Total Insecurity scales than non-biological parents.

### Measurement invariance

The next steps tested measurement invariance between fathers (*N* = 320) and mothers (*N* = 361) of the 12-item model that was empirically derived from the previous steps. Each step had more stringent requirements for measurement invariance. Interpretations of the results are based on the description of the different types of factorial measurement invariance provided by Gregorich [36].

Model 3 tested configural invariance and showed a good fit to the data. This indicates that the three common factors (i.e., the three attachment types) are associated with the same items across groups, that is, fathers and mothers. Model 4 tested metric invariance, which showed a good fit to the data, and no worse fit than the previous model, *X*2 (9) = 7.405, *p* = .595, ΔCFI = .000, indicating that the three common factors have the same meaning across fathers and mothers, and are not differentially affected by extreme response styles (i.e., never-always), or the tendency to avoid extreme answers. Model 5a tested scalar invariance, and had a significantly worse fit than the previous model, *X*2 (9) = 32.228, *p* < .001, Δ CFI = .009. This result implies systematically higher- or lower-valued item response between the groups, causing different group means for fathers and mothers (see Table 3). Consequently, mothers and fathers cannot be compared on their mean scores on the three attachment types. Model 5b is a test of partial scalar invariance, which shows that the model did not fit the data worse than model 4, *X*2(4) = 2.717, *p* = .606, Δ CFI = .001, if the intercepts of 5 items were free to vary: disorganization (7 and 18), avoidance (14) and ambivalence (10 and 16). This implicates that the scores on the other items can be compared between fathers and mothers. Model 6a of strict invariance did fit the data significantly worse than model 5a (*X*2(12) = 25.310, *p* = .013, Δ CFI = .005). This indicates that the observed variances of mothers and fathers are not equal, and cannot be compared in a meaningful way. Finally, model 6b provides a test of partial strict invariance, which did not fit the data worse than model 5b (*X*2 (11) = 14.750, *p* = .194, Δ CFI = .002), if the residual variance of item 7 was free to vary. This implies that the observed variances of mothers and fathers can be compared on the other items.

**Table 3:** Means and SDs of Fathers (*n* = 320) and Mothers (*n* = 361) on the items of the AISI 6–12 years

	Fathers		Mothers		
	*M*	*SD*	*M*	*SD*	Paired *t*^a^
*Disorganized (Type D)*					
1. Does your child try to force you to do what he/she wants?	2.41	1.08	2.66	1.23	2.894**
7. Does your child argue with you if things do not turn out the way he/she expects?	3.31	1.38	3.47	1.40	1.159
12. Is your child excessively determined to decide everything for him/herself?	3.20	1.45	3.40	1.51	1.398
18. Does your child become angry with you quickly?	2.54	1.10	2.96	1.30	5.489***
*Ambivalent (Type C)*					
6. Does your child always stay close to you?	2.57	1.10	2.73	1.23	1.966
10. Is your child over-concerned when you are upset or unwell?	2.62	1.37	2.68	1.43	0.463
16. Does your child keep a close eye on you while you do things in and around the house?	2.30	1.10	2.71	1.30	4.140***
20. Does your child need you to reassure him/her that he/she is doing something right?	3.18	1.27	3.48	1.38	3.608***
*Avoidant (Type A)*					
8. Does your child let you comfort him/her if he/she is in pain, frightened or upset (R)?	4.53	1.43	4.49	1.49	0.765
9. Does your child ask for help with problems (R)?	3.25	1.23	3.30	1.34	0.677
11. Does your child make good contact with you after you have been away for a short period of time (R)?	4.74	1.30	4.70	1.40	0.557
14. Is your child able to enjo) y contact with you (R)?	4.37	1.19	4.53	1.25	2.701**

*Note.* ^a^*N* = 309. *p < .05; **p < .01; ***p < .001

### Reliability of the scales

We have tested the Ordinal and Cronbach’s alphas for the 12-item version of the AISI. Ordinal alpha was .67 (Cronbach’s α = .65) for ambivalent attachment, for avoidant attachment .82 (Cronbach’s α = .80), and for disorganized attachment .86 (Cronbach’s α = .85). For fathers, the ordinal alpha for ambivalent attachment was .66 (Cronbach’s α = .63), for avoidant attachment .77 (Cronbach’s α = .77), and for disorganized attachment .81 (Cronbach’s α = .82). For mothers, the ordinal alpha was .67 (Cronbach’s α = .6), .83 (Cronbach’s α = .82), and .87 (Cronbach’s α = .87), respectively. The internal consistency of the ambivalent scale was therefore marginal; the other two scales had a good internal consistency.

## Discussion

The present study aimed to examine the internal structure and reliability of the AISI 6–12 years [18] in a clinical sample of primarily Dutch adoptive and foster care families with children aged 6 to 12. Multilevel CFA resulted in a 12-item instrument that purports to measure the parents’ perception of the quality of attachment relationships with their children. The three subscales Avoidant (Type A), Ambivalent (Type C), and Disorganized (Type D) attachment relationships each consist of four items. The 12-item instrument has the same factor structure (configural invariance) and the same meaning (metric invariance) for fathers and mothers. Further, evidence was found for partial scalar and strict invariance, which means that mothers and fathers showed similar mean scores and observed variances for most items, but not for all. Mothers tended to report systematically more problems on certain items than did fathers. The internal consistency was good for the Avoidant (Type A) and Disorganized (Type D) subscales, and marginally sufficient for the Ambivalent (Type C) scale.

In total, we have removed eight items of the original 20-item scale. The 12-item AISI 6–12 years had improved factor structure, similar reliability estimates, and a high correlation with the original 20-item scale. In addition, we believe that the face validity of the AISI 6–12 years has been improved by removing eight items. For example, the four removed items of the Avoidant (Type A) subscale all focused on whether a child is relaxed in the presence of the parent or in physical contact with the parent (e.g.*, Is your child happy and playful in your presence (R)? Does your child enjoy being cuddled by you (R)? Does your child respond well and remain relaxed when you touch him/her (R)?*). Tension or stress experiences by the child in contact with the parent are not exclusively characteristic of avoidant attachment, but may also be present in ambivalent or resistant [43], and especially disorganized attachment relationships [44]. Therefore, it makes sense that these items had to be removed from the avoidant subscale. In addition, the Disorganized item *Does your child stay in control when playing with you?* was removed. Possibly, there is a shift in middle childhood from play with parents to play with peers, and therefore it is harder for parents to answer. Moreover, the formulation of the item is rather abstract, and therefore harder for parents to recognize.

It is remarkable that in the Ambivalent (Type C) subscale the two most “basic” items that should represent ambivalent attachment patterns (i.e., *Does your child want to be left alone and simultaneously seeks contact with you?* and *Does separation from you cause overly strong emotional reactions in your child?*) had to be removed in the multilevel CFA steps in order to obtain a satisfactory model fit. There are several explanations for this result. First, it is possible that the items from the factor solution do not provide a good representation of ambivalent attachment patterns, and that the subscale Ambivalent (Type C) does not have sufficient construct and/or content validity. A second explanation may be that parents find it difficult to detect ambivalence in the interactions with their child. The ambivalence of resistance and proximity-seeking at the same time makes it very difficult to correctly interpret the intentions of behaviors. Parents may notice the resistance (which may in fact cause an emotional response in the parents as well), but fail to notice the simultaneously occurring contact-seeking behaviors. More extensive research on the expression of (insecure) attachment behaviors in middle aged children may shed light on this issue. Finally, it may be that ambivalent attachment is expressed differently in middle childhood compared to early childhood [7]. Ainsworth, Blehar, Waters and Wall [8] described extreme distress during separation from the mother and simultaneous occurrence of resistance and contact-seeking behavior in ambivalently attached young children and infants. However, Bosmans and Kerns [7] note that it is not so much the *proximity* that middle school aged children need, but the *availability* of the attachment figure. Separation from the attachment figure would then not necessarily cause distress in children with ambivalent attachment patterns if the caregiver is still psychologically available in alternative ways in the child’s perception, or perhaps physically available, for instance by means of mobile phones or other devices. Also, ambivalently attached middle school children have been shown to rather display their distress in a more regulated, passive-aggressive and manipulative way rather than through an overly emotional reaction [28, 43].

This study showed that the AISI 6–12 years meets the demands of the most important types of measurement invariance across fathers and mothers. The factor structure (configural invariance) and meaning (metric invariance) was similar for fathers and mothers, implicating that based on the internal structure, the AISI 6–12 years can be filled in by both fathers and mothers. However, we found that for certain items mothers reported structurally more attachment problems then fathers, implicating only partial scaler invariance. To our knowledge, there are almost no indications from previous studies that children have a less secure attachment relationship with their mothers than with their fathers [45], although George, Cummings and Davies [46] showed that children can exhibit a different pattern of attachment with their father and mother and the relation between sensitivity and attachment is somewhat stronger in mothers than in fathers [47]. Moreover, fathers may show differences in the way they express their (play) sensitivity towards the child, which has been shown to be related to their child’s development of attachment over time [48]. Literature on the interparental agreement of behavior showed that mothers reported systematically more internalizing, externalizing and total problem behaviors than did fathers [49], possibly because mothers perceive similar behavior as more problematic than fathers and feel more responsible for developmental problems in their children [50]. As the AISI 6–12 years intents to measure the parental *perception* of the attachment relationship with the child, this seems to be a valid explanation for differences between fathers and mothers. Because we only found partial scaler and strict invariance, mean scale scores and observed variances cannot be compared across mothers and fathers in a meaningful manner. For both scientific and clinical use, it is important to include the father’s perspective on the attachment relationship with his child. In order to make the AISI 6–12 years suitable for both parents, it seems therefore important to create different cut-off scores for fathers and mothers.

The reliability of the Ambivalent (Type C) subscale was questionable for use in clinical practice (α = .65). The reliability of the scores could be underestimated because the scale only consists of 4 items [51]. Moreover, the items of the scale appear to measure multiple dimensions of ambivalent attachment patters. The items relate to both the need for proximity/availability and self-efficacy of ambivalent attachment (i.e., *Does your child need you to reassure him/her that he/she is doing something right?*) [52]. This may explain the marginal reliability of the Ambivalent (Type C) subscale. Future studies could test this explanation.

This study has several limitations that need to be mentioned. First, this study assessed only the internal structure and reliability of the AISI 6–12 years. Other dimensions of validity (such as concurrent, predictive and convergent validity) have not been tested. Second, in the analyses to test measurement invariance across fathers and mothers, it was not possible to account for the multilevel structure of the data (i.e., children of the same family were nested within the same mother-father dyads) at the same time. We therefore followed a hierarchical procedure to examine the internal structure of the AISI, first accounting for dependency by means of taking the nested structure of the data into account, and subsequently testing measurement invariance of the best fitting multi-level factor model. Finally, the internal structure and measurement invariance of the AISI 6–12 years was tested in a clinical sample, which consisted mostly of adoptive and foster care families. The findings of the current study may therefore be limited generalizable to other samples, such as non-clinical samples. Moreover, the sample consisted of at risk children of biological parents, next to non-biological parents. Including biological parents made the sample more heterogeneous, but also increased external validity.

Taking the limitations of the current study into consideration, this study contributes to the literature on attachment in middle childhood, and offers interesting implications for future research and clinical practice. First, the current study provides suggestions for further studies on (the expression of) attachment in middle childhood. That is, there are some clues from previous studies that the expression of insecure attachment problems in middle childhood is different than the expression in early childhood [28, 43, 53], and therefore, taking measurements of attachment in younger children as a starting point of measurements of attachment for middle childhood may not be appropriate. However, we did find evidence for the three factor structure of insecure attachment (Type A, C, and D) in middle childhood, which implicates that the insecure attachment classifications that are used in infancy, early childhood, and adolescence have also meaning in middle childhood. The literature on attachment insecurity in middle childhood is somewhat underdeveloped, in particular due to lack of reliable and valid measurement instruments [7, 14]. We therefore follow the suggestion of Bosmans and Kerns [7] that future research should focus on identifying age-related changes in insecure attachment patterns in order to create instruments that can measure the quality of attachment relationships in middle childhood.

A second implication is that the current study paves the way for the assessment of parental perceptions of the quality of the attachment relationship with the child. Based on the internal structure and reliability of the AISI 6–12 years, it seems that parental reports can be a valuable source of information in assessing attachment problems in middle childhood. Using questionnaires for parents is a rather practical approach, with yields outcomes that are easy to interpret. Other measures that are currently used to assess attachment patterns in middle childhood (such as observations, projective doll play interviews, and self-report interviews) are rather time consuming, require extensive training, or are less suitable for children in the early stages of middle childhood or children with learning disabilities [14]. Especially for child protective services, primary care providers, or in intake procedures the AISI 6–12 years is potentially useful in the initial screening of attachment related problems from the perspective of parents themselves. Instruments assessing the parents’ perspective on the attachment relationship with their child can offer interesting clinical information, but cannot (solely) be used as a diagnostic instrument. So, if scores on the AISI 6–12 years are elevated, and after additional evidence of attachment related problems in a standard clinical interview or based on case file information, families could be referred to specialists in diagnosing attachment problems for a more comprehensive observation-based assessment of attachment problems and, if necessary, to attachment-based interventions (see for example [16, 53, 54]). Moreover, the AISI 6–12 years can be used as a point of engagement to discuss attachment experiences of parents with their children in diagnostics and treatment, which could strengthen the possibilities for attachment-based interventions.

The final implication of this study is that the AISI 6–12 years needs further research and improvement before it can be validly and reliably used in scientific studies and clinical practice. The AISI 6–12 years [18] was based on the AISI 2–5 years [14, 17], although items were slightly changed on account of age-appropriateness. The AISI 6–12 years may need even further alterations to make it more appropriate for middle childhood, for example, by including items on passive-aggressive, self-determining, and manipulative behavior in the Ambivalent (Type C) scale [43], and items on role reversal in the Disorganized (Type D) scale [23].

Future studies should examine other sources of validity indications of the AISI 6–12 years, such as convergent and concurrent validity by comparing scores of the AISI 6–12 years with other measures that examine attachment patterns in middle childhood, and with measures that assess psychopathology and adjustment problems [5, 53, 55]. Although, Bosmans and Kerns [7] argue that different measurement strategies tap into different components or aspects of the child-parent attachment relationship, and therefore do not necessarily have to correlate in order to be valid (see also [56]). The AISI 6–12 years aims to measure the parents’ perspective on the quality of the attachment relationship with their child. Therefore, it is valuable to compare results of the AISI 6–12 years with classifications of the Adult Attachment Interview (AAI [10]), an instrument aimed at assessing attachment representations of parents and other caregivers. The AAI is a strong predictor of the quality of the attachment relationships between parents and children [57]. The pathway of intergenerational transmission of attachment goes from parents’ attachment representations, through parental sensitivity [58] and parents’ mentalizing abilities to understand the internal states of their child (i.e., mind mindedness [59]). The AISI might cover a component relevant for the intergenerational transmission of attachment, that is, the parents’ interpretation of their child’s behavior from an attachment perspective. However, cut-off scores and the power of the AISI 6–12 years to discriminate between children with and without attachment insecurity need to be known before this instrument can be used in clinical practice.

## Conclusions

In conclusion, this study showed that the internal structure of parent reports on the perception of the quality of the relationship with their child can be sufficient and largely measurement invariant across mothers and fathers. Even though the AISI 6–12 years needs further development and more research on (insecure) attachment patterns in middle childhood and the validity of the AISI 6–12 years is necessary, we pledge for the use of parent reports of the attachment relationships apart from other assessment methods. Parent reports offer valuable insights into parental representations and the behavior of children. In the end, parents do not rely on a single observation, but know their children’s behavior in different contexts and for an extended period of time. Additionally, the parent’s perception of their child may influence their parenting behavior, which impacts on the way a child develops, including the child’s attachment. Insecure attachment relationships are predictive of all types of developmental problems in life [4, 5, 53, 55]. Therefore, parental reports provide in the need of clinical practice for straightforward instruments that can be used in the screening of attachment related problems and the ability to include parental perceptions in attachment research.

## Additional file

Additional file 1: Supplementary File AISI 6–12. Items of the AISI 6–12 years. This file contains the items of the AISI 6–12 years. (DOCX 15 kb)

## Abbreviations

AAI: Adult Attachment Interview
AISI: Attachment Insecurity Screening Inventory
CFA: Confirmatory Factor Analyses
