# Supplementary material for: Internal structure and reliability of the Attachment Insecurity Screening Inventory (AISI) for children age 6 to 12
Source: BMC Psychiatry. 2018 Feb 5;18:30. doi: 10.1186/s12888-018-1608-z (PMC5800067; doi:10.1186/s12888-018-1608-z)
Supplement: Additional file 1: Supplementary File AISI 6–12. — Items of the AISI 6–12 years. This file contains the items of the AISI 6–12 years. (DOCX 15 kb) [file 12888_2018_1608_MOESM1_ESM.docx]

**Supplementary File AIS 6-12**

*Attachment Insecurity Screening Inventory (AISI) 6-12 years: 20-item Parental Report (1 = never; 2 = sometimes; 3 = regularly; 4 = often; 5 = very often; 6 = always)*

1. Does your child try to force you to do what he/she wants?
2. Is your child excessively docile and obedient?
3. Does your child respond well and remain relaxed when you touch him/her (R)?
4. Does your child stay in control when playing with you?
5. Does your child enjoy being cuddled by you (R)?
6. Does your child always stay close to you?
7. Does your child argue with you if things do not turn out the way he/she expects?
8. Does your child let you comfort him/her if he/she is in pain, frightened or upset (R)?
9. Does your child ask for help with problems (R)?
10. Is your child over-concerned when you are upset or unwell?
11. Does your child make good contact with you after you have been away for a short period of time (R)?
12. Is your child excessively determined to decide everything for him/herself?
13. Does separation from you cause overly strong emotional reactions in your child?
14. Is your child able to enjoy contact with you (R)?
15. Does your child want to be left alone and simultaneously seeks contact with you?
16. Does your child keep a close eye on you while you do things in and around the house?
17. Does your child hug or cuddle you spontaneously (R)?
18. Does your child become angry with you quickly?
19. Is your child happy and playful in your presence (R)?
20. Does your child need you to reassure him/her that he/she is doing something right?

*Note*. Avoidance subscale items: 3, 5, 8, 9, 11, 14, 17, 19, Ambivalence/resistance subscale items: 2, 6, 10, 13, 15, 16, 20, Disorganization subscale items: 1, 4, 7, 12, 18; (R) = reversely coded.
